# The association of serum CHI3L1 levels with the presence of Kawasaki disease

**DOI:** 10.1038/s41598-025-91935-8

**Published:** 2025-03-05

**Authors:** Jialin Zou, Xu He, Wenjuan Li, Yan Duan, Bin Liu, Jun Jiang, Dinghua Luo, Jian Zhao, Gang Li

**Affiliations:** 1 Department of Pediatrics, The Affiliated Hospital of Southwest Medical University, No. 25 Taiping Street, Luzhou, Sichuan China; 2 Sichuan Clinical Research Center for Birth Defects, Sichuan, China; 3 Department of General Surgery (Thyroid Surgery), The Affiliated Hospital of Southwest Medical University, Sichuan, China; 4 Metabolic Vascular Diseases Key Laboratory of Sichuan Province, Sichuan, China

**Keywords:** Kawasaki disease, Chitinase 3-like protein 1, Biomarker, Biochemistry, Immunology, Biomarkers, Rheumatology, Risk factors

## Abstract

Chitinase 3-like protein 1(CHI3L1) has been found to be a biomarker for inflammatory diseases, but the diagnostic value of Kawasaki disease (KD) is not investigated. A total of 180 subjects, including 80 KD patients, 70 febrile controls and 30 healthy controls were recruited. Serum of CHI3L1 were measured with an enzyme-linked immunosorbent assay. The correlation between CHI3L1 and clinical parameters was assessed by Spearman correlation coefficient. Multiple logistic regression was employed to investigate the association between CHI3L1 and the incidence of KD. The diagnostic power was evaluated with the receiver operating characteristic curve analysis. Serum CHI3L1 levels in the KD group were significantly higher than those in the fever control group and healthy group. Compared with fever patients, both patients with incomplete KD and complete KD had higher serum CHI3L1 levels. Serum CHI3L1 levels were positively associated with white blood cell counts, neutrophils, platelet, erythrocyte sedimentation rate, C-reactive protein, alanine aminotransferase and the incidence of KD, and negatively associated with hemoglobin, aspartate aminotransferase and albumin. High CHI3L1 tertiles was significantly associated with the high incidence of KD in the unadjusted or adjusted models. Analysis of the Receiver operating characteristic curves, it was showed that the area under the curve was 0.908, with sensitivity of 0.838 and specificity of 0.8 for continuous CHI3L1, and was 0.884 for categorical CHI3L1, with sensitivity of 0.938 and specificity of 0.643 to distinguish all types of KD, respectively. CHI3L1 had the AUC of 0.901, with sensitivity of 0.826 and specificity of 0.8, and had the area under curve of 0.952, with sensitivity of 0.818 and specificity of 0.971 to discriminate complete KD and incomplete KD from febrile diseases, respectively. Serum of CHI3L1 may be a novel and reliable biomarker for the diagnosis of KD.

## Introduction

Kawasaki disease (KD) is an acute inflammatory disorder primarily affecting infants and young children, leading to the development of coronary artery lesions (CAL) and consequently being recognized as the most prevalent cause of acquired heart disease in children in developed nations. Recent research indicates a significant prevalence of incomplete KD (iKD) both domestically and internationally^1^. A nationwide survey in Japan conducted from 2013 to 2016 revealed that 22% of cases were classified as iKD. Additionally, studies suggest a rising incidence of iKD in China^1^. Children with iKD often experience delayed diagnosis, particularly in infants and older children over 5 years old, which can increase the risk of coronary artery lesions (CAL)^2^. Despite being identified over 60 years ago by Dr. Tomisaku Kawasaki, the diagnosis of KD continues to heavily rely on clinical symptoms due to the absence of definitive biomarkers, potentially resulting in KD cases being overlooked. Further exploration of potential laboratory diagnostic biomarkers is great importance for the timely diagnosis and therapy of KD.

Previous studies have extensively investigated potential markers for KD. While various inflammatory markers such as white blood cell (WBC) count, C-reactive protein (CRP)^3^, IL-6^4^ and prokineticin 2^5^ have shown potential for diagnosing KD either in combination with clinical symptoms/other inflammatory parameters or independently, they generally lack specificity for the disease. Chitinase 3-like protein 1 (CHI3L1), also known as YKL-40 in humans, is a secreted glycoprotein with multiple functions that is expressed in various cell types, including endothelial cells, activated macrophages, chondrocytes, neutrophils, fibroblasts, synovial and various tumor cells^6^. It has been established that CHI3L1 plays a significant role in the regulation of various essential biological processes, including inflammatory activation, oxidative damage, and apoptosis^7^, as well as in immune cell function^6^. Additionally, it has been implicated in inflammatory diseases^7–10^. Recent research has shown that CHI3L1 serves as a marker for several inflammatory conditions, including atherosclerosis, acute coronary syndrome or stable coronary artery disease^11^, rheumatoid arthritis^12^ and system lupus erythematosus^13^. Prior research has indicated an elevation of CHI3L1 in both serum and coronary arteries in a KD mouse model induced by *lactobacillus casei* cell wall extract^14^. Additionally, a study by KY et al. reported an increase in serum CHI3L1 levels in KD^15^. Nevertheless, the sample size was limited and the diagnostic potential of CHI3L1 in the acute phase of KD remains incompletely understood.

Building upon these findings, the current study seeks to validate the diagnostic utility of CHI3L1 in the acute phase of KD.

## Materials and methods

### Patients

80 patients with acute phase of KD were recruited between Jun 2021 and December 2023 from the Affiliated Hospital of Southwest Medical University. Diagnosis of complete KD (cKD) and iKD met the standard of the AHA 2017^16^. Exclusion criteria: other immune diseases; treatment with corticosteroid; treatment of IVIG or aspirin before hospital during the current course of disease; metabolic diseases; liver and kidney diseases. Simultaneously, 70 patients with common fever who were admitted to hospital were selected and 30 healthy children were recruited during the same time period. These common fever patients had infection and were diagnosed with upper or lower respiratory tract infections, encephalitis and sepsis. This study was approved by the Ethics Committee of the Affiliated Hospital of Southwest Medical University (KY2024193) and the written informed consent was obtained from each participant’s guardian. The informed consent for the collection of serum samples in our study were obtained from each participant’s guardian. All procedures were performed in accordance with the ethical standards of the 1964 Helsinki Declaration and its later amendments.

Clinical laboratory data for patients with KD were collected before initial infusion of intravenous immunoglobulin (IVIG), including demographic characteristics, WBC, neutrophils counts (NEU), lymphocytes counts (LYM), hemoglobin (Hb), platelet counts (PLT), CRP, erythrocyte sedimentation rate (ESR), aspartate aminotransferase (AST), albumin (ALB), alanine aminotransferase (ALT), and procalcitonin (PCT).

### Sample collection and measurement of serum CHI3L1

Serum samples were collected from participants with KD before infusion of IVIG and serum samples of patients with common fever were obtained before treatment. All samples were centrifuged at 1000 rpm for 10 min, and then stored at -80℃ until testing. The concentration of CHI3L1(CUSABIO, NO.CSB-E13608h, USA) was measured with an enzyme-linked immunosorbent assay (ELISA) according to the manufacturer’s instructions. According to the CHI3L1 levels, participants with KD and common fever were divided into three equal parts: T1 group (≤ 2849 pg/ml, *n* = 50), T2 group (2850–6021 pg/ml, *n* = 50) and T3 group (≥ 6022 pg/ml, *n* = 50).

### Statistical analysis

Statistical analysis was performed with SPSS version 24.0 (IBM Corp., Armonk, NY, USA), GraphPad Prism 9 (GraphPad Software, Inc., San Diego, CA, USA) and Python software (statsmodels 0.11.1). Continuous variables are presented as median with interquartile range, and categorical variables are presented as number (%). Categorical variables were analyzed by chi-square tests, continuous variables were analyzed by the t test or Mann–Whitney U test or one-way analysis of variance, and correlation analysis was analyzed by the Spearman test. To investigate the relationship CHI3L1and KD prevalence, logistic regression analysis was employed. We established three models: Model 1, unadjusted; Model 2, adjusted for gender; and Model 3, adjusted for gender, WBC, NEU, Hb, PLT, ESR, CRP, ALT and ALB. The restricted cubic spline (RCS) was employed to explore the dose-response association of continuous CHI3L1 and the incidence of KD. Receiver operating characteristic (ROC) curve analysis was performed to assess the diagnostic ability of CHI3L1. Values of *p* < 0.05 were considered to indicate statistical significance.

## Results

### Clinical characteristics of patients with KD

In a cohort of 80 patients diagnosed with KD, 54 were male and 26 were female, with a median age of 35 months (range: 16.8–49.0 months). Of these patients, 69 had cKD and 11 had iKD. Compared to both healthy and fever control groups, the KD group exhibited significantly elevated WBC, NEU, PLT, CRP, ESR, PCT, and ALT, as well as decreased levels of Hb, ALB, and AST (all *p* < 0.05). There were no significant differences in BMI, age, gender, or LYM between the groups (all *p* > 0.05) (Table 1).

**Table 1:** Baseline characteristics participants.

Variables	Overall (*N* = 180)	Healthy control ( *N* = 30)	Fever (*N* = 70)	KD (*N* = 80)	*p*-value
Male, n (%)	105 (58)	16 (53)	35 (50)	54 (68)	0.079
Age (Months)	33.5 (16.8–49.0)	40.5 (24.3–52.8)	30.0 (16.0-40.8)	35.0 (16.8–49.0)	0.415
BMI (Kg/m^2^)	15.7 (14.4–17.2)	15.4 (14.3–17.0)	15.6 (14.4–16.8)	15.9 (14.4–17.6)	0.393
WBC(×10^9^/L)	10.6 (7.5–15.3)	7.4 (6.1–8.7)	8.9 (7.2–12.3)	14.1 (11.3–17.3)	< 0.001
NEU(×10^9^/L)	6.6 (3.7–10.5)	3.3 (2.3-4.0)	5.2 (3.6–8.2)	9.8 (7.7–11.7)	< 0.001
LYM(×10^9^/L)	2.9 (1.9–3.9)	3.3 (2.4–3.9)	2.5 (1.7–3.8)	2.9 (2.0–4.0)	0.167
Hb (g/L)	118.0 (110.0-126.3)	125.5 (118.0-129.8)	119.0 (114.0-126.8)	112.5 (107.8–121.)	< 0.001
PLT(×10^9^/L)	326.5 (263.0-404.8)	299.0(271.0-364.3)	281.5 (235.8-359.3)	374.0 (302.8-455.8)	< 0.001
ESR (mm/h)	34.0 (14.0-60.3)	11.5 (7.0-16.8)	20.0 (11.3–34.8)	66.5 (43.3–85.5)	< 0.001
CRP(mg/L)	24.7 (6.5–62.1)	2.4 (1.3-5.0)	11.6 (7.3–55.4)	52.0 (26.1–89.6)	< 0.001
PCT (ng/ml)	0.320 (0.01–0.78)	0.05 (0.03–0.29)	0.21 (0.02–0.55)	0.53 (0.29–1.84)	< 0.001
ALB(g/L)	42.3 (38.3–44.7)	44.2 (42.9–45.7)	43.9 (42.3–46.0)	38.30(35.8–40.6)	< 0.001
AST(U/L)	33.8 (27.4–42.8)	34.1 (29.9–37.9)	37.3 (31.6–48.5)	30.4 (24.6–40.0)	0.004
ALT(U/L)	17.7(12.9–30.5)	14.5 (11.9–18.0)	15.7 (12.7–20.1)	24.9 (16.7–75.6)	< 0.001

*WBC* white blood cell counts, *NEU* neutrophils, *LYM* lymphocytes, *Hb* hemoglobin, *PLT* platelet, *CRP* C-reactive protein, *ESR* erythrocyte sedimentation rate, *AST* aspartate aminotransferase, *ALB* albumin, *ALT* alanine aminotransferase, *PCT* procalcitonin.

### Serum levels of CHI3L1 in all participants

**Fig. 1:**
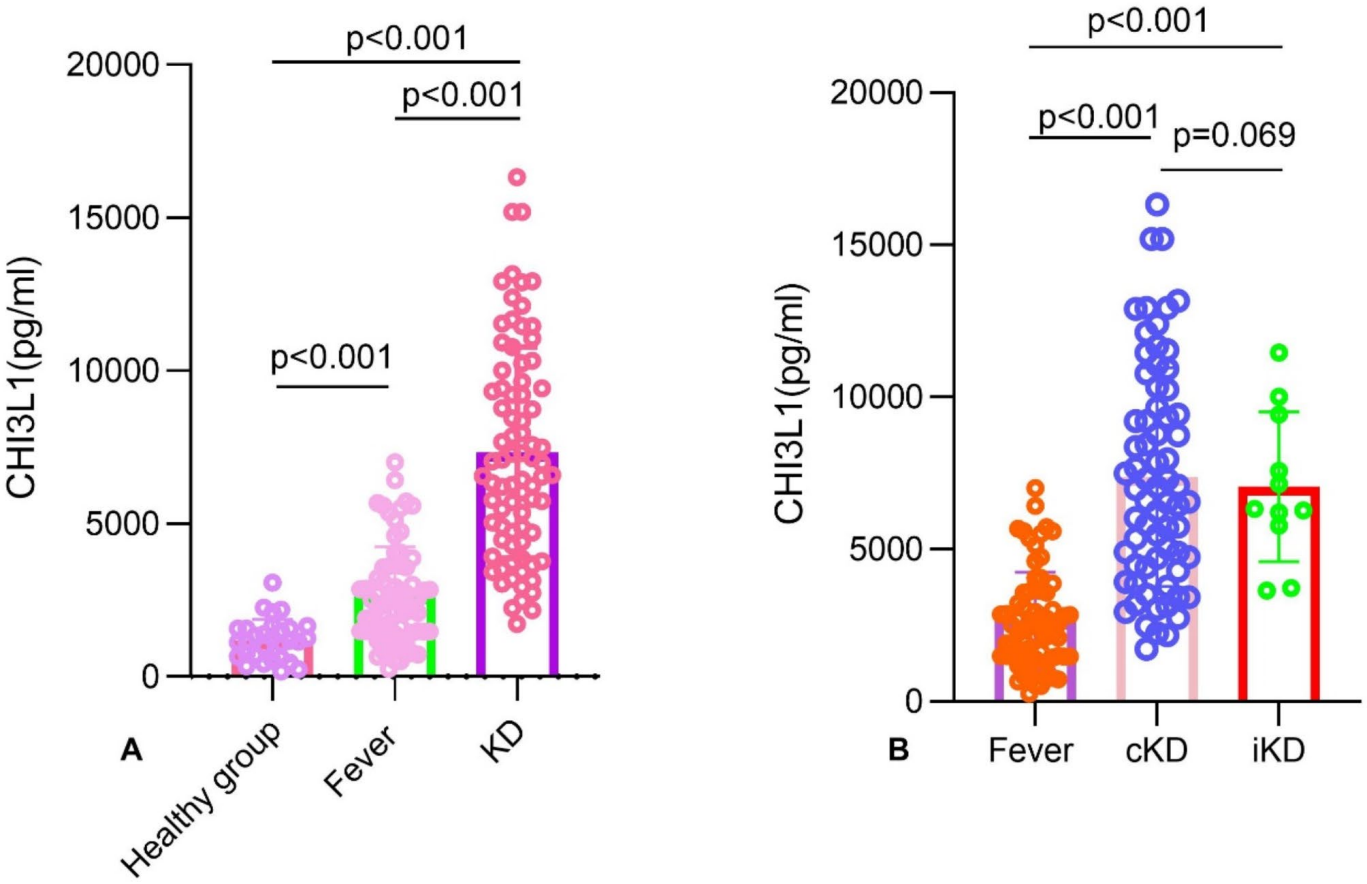
Serum of CHI3L1 in KD, fever and healthy control.

Serum of CHI3L1 levels were significantly higher in KD patients compared to both the fever control and healthy groups (*P* < 0.001), as illustrated in Fig. 1A. In comparison to the fever group, patients with iKD and cKD exhibited significantly elevated serum CHI3L1 levels (*P* < 0.001) (Fig. 1B).

### Participant characteristic according to the tertiles of CHI3L1 among patients with KD or fever

**Fig. 2:**
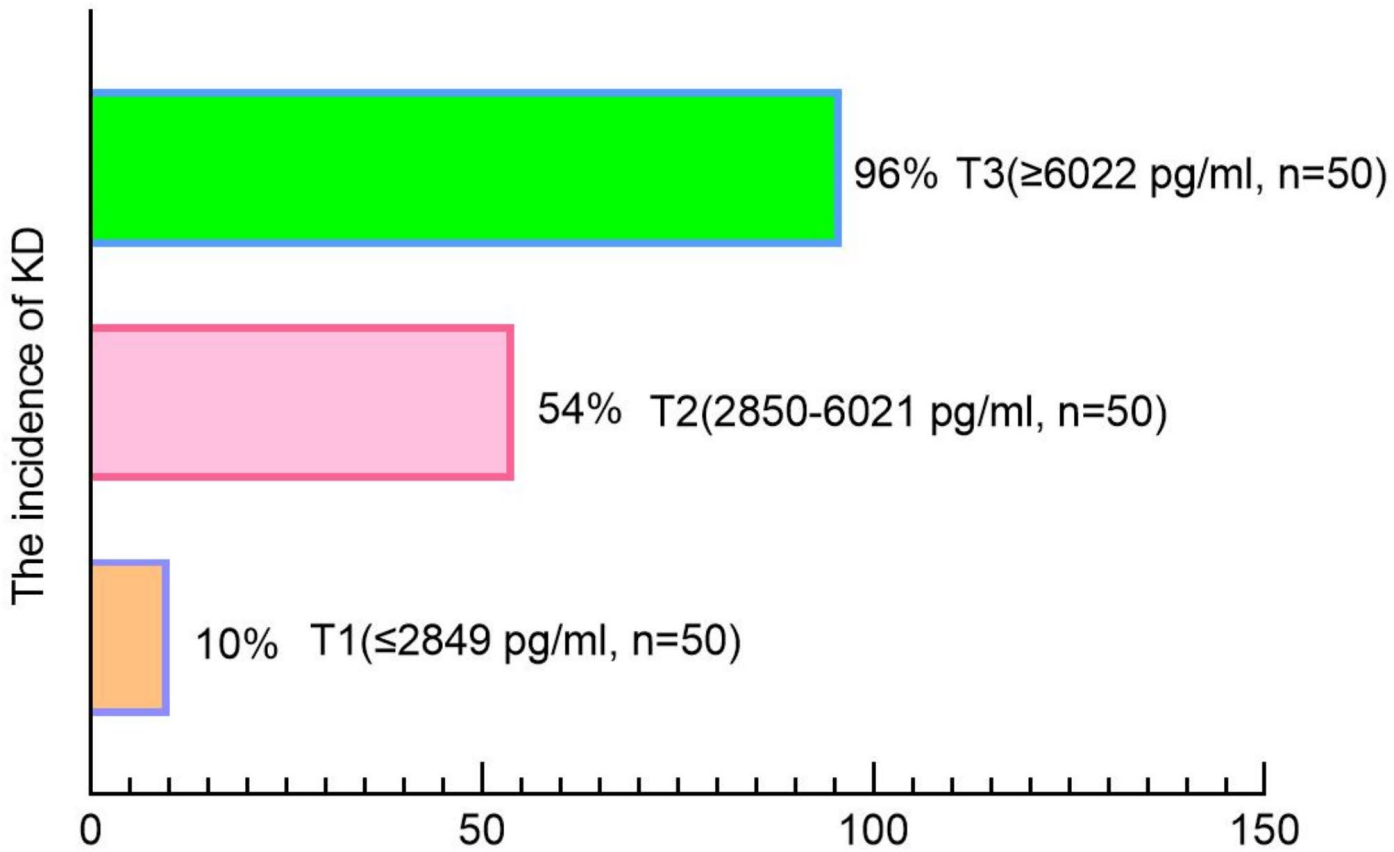
The incidence rates comparison of KD in the three groups.

The baseline characteristics of patients with KD or fever based on the tertiles of CHI3L1 levels were presented in Table 2. Among the three groups, individuals with higher CHI3L1 levels displayed elevated WBC, NEU, PLT, ESR, CRP, and ALT, as well as decreased levels of Hb and ALB, and were more likely to be male (all *p* < 0.05). As depicted in Fig. 2, there was a significant association between higher serum CHI3L1 levels and increased incidence of KD (*p* < 0.01).

**Table 2:** Characteristics of study population according to quartile of CHI3L1 among patients with KD or fever.

Clinical and Biochemical Characteristics	Total Population (*N* = 150)	T1 (*N* = 50)	T2 (*N* = 50)	T3 (*N* = 50)	*p*-value
Demographic Variables					
Male, n (%)	89 (59)	27 (54)	25 (50)	37 (74)	0.033
Age (Months)	33.00 (16.00–48.75)	33.00 (16.25-49.00)	33.50 (17.50–45.50)	33.50 (16.00-48.75)	0.89
BMI (Kg/m^2^)	15.71 (14.41–17.27)	15.61 (14.40-16.94)	15.60 (14.40-16.69)	16.48 (15.11–17.72)	0.2
Biochemical Variables					
WBC(×10^9^/L)	12.15 (8.45–16.22)	9.48 (7.25–13.48)	13.22 (8.33–15.65)	13.77 (10.95–17.27)	0.01
NEU(×10^9^/L)	8.34 (4.59–10.73)	5.69 (3.73–9.66)	8.34 (5.05–10.53)	9.60 (7.15–11.46)	0.009
LYM(×10^9^/L)	2.76 (1.84–3.89)	2.48 (1.67–3.97)	2.87 (1.90–3.61)	3.00 (2.10–4.47)	0.28
Hb (g/L)	117.0 (109.0-125.0)	120.0 (113.25-126.75)	115.5 (108.25-123.75)	111.5 (108.0-121.0)	0.015
PLT(×10^9^/L)	331.0 (263.0-417.0)	290.5 (234.0-365.50)	353.00 (267.25–414.5)	358.5 (300.5–473.0)	0.002
ALB(g/L)	41.55 (37.20–44.10)	43.65 (41.55-47.00)	42.20 (38.28–44.57)	38.25 (35.58–40.48)	< 0.001
AST(U/L)	33.80 (27.13–45.55)	35.75 (31.40-48.48)	35.44 (27.38–44.65)	30.20 (24.75-40.00)	0.054
ALT(U/L)	18.25 (13.30–33.00)	15.45 (12.83–19.88)	18.0 (12.88-33.0)	30.30 (17.18–85.48)	< 0.001
Inflammatory Markers					
PCT (ng/ml)	0.39 (0.12–1.03)	0.26 (0.06–0.63)	0.37 (0.11–0.97)	0.45 (0.24–1.77)	0.066
ESR (mm/h)	39.50 (20.25–69.75)	18.50 (8.25–33.75)	40.50 (24.25–77.50)	56.00 (41.00-78.75)	< 0.001
CRP(mg/L)	37.06 (10.44–70.74)	21.41 (7.94–58.30)	31.72 (10.36–64.95)	54.29 (27.07–82.52)	0.007

*WBC* white blood cell counts, *NEU* neutrophils, *LYM* lymphocytes, *Hb* hemoglobin, *PLT* platelet, *CRP* C-reactive protein, *ESR* erythrocyte sedimentation rate, *AST* aspartate aminotransferase, *ALB* albumin, *ALT* alanine aminotransferase, *PCT* procalcitonin.

### Correlation of serum CHI3L1 levels with the laboratory parameters

**Fig. 3:**
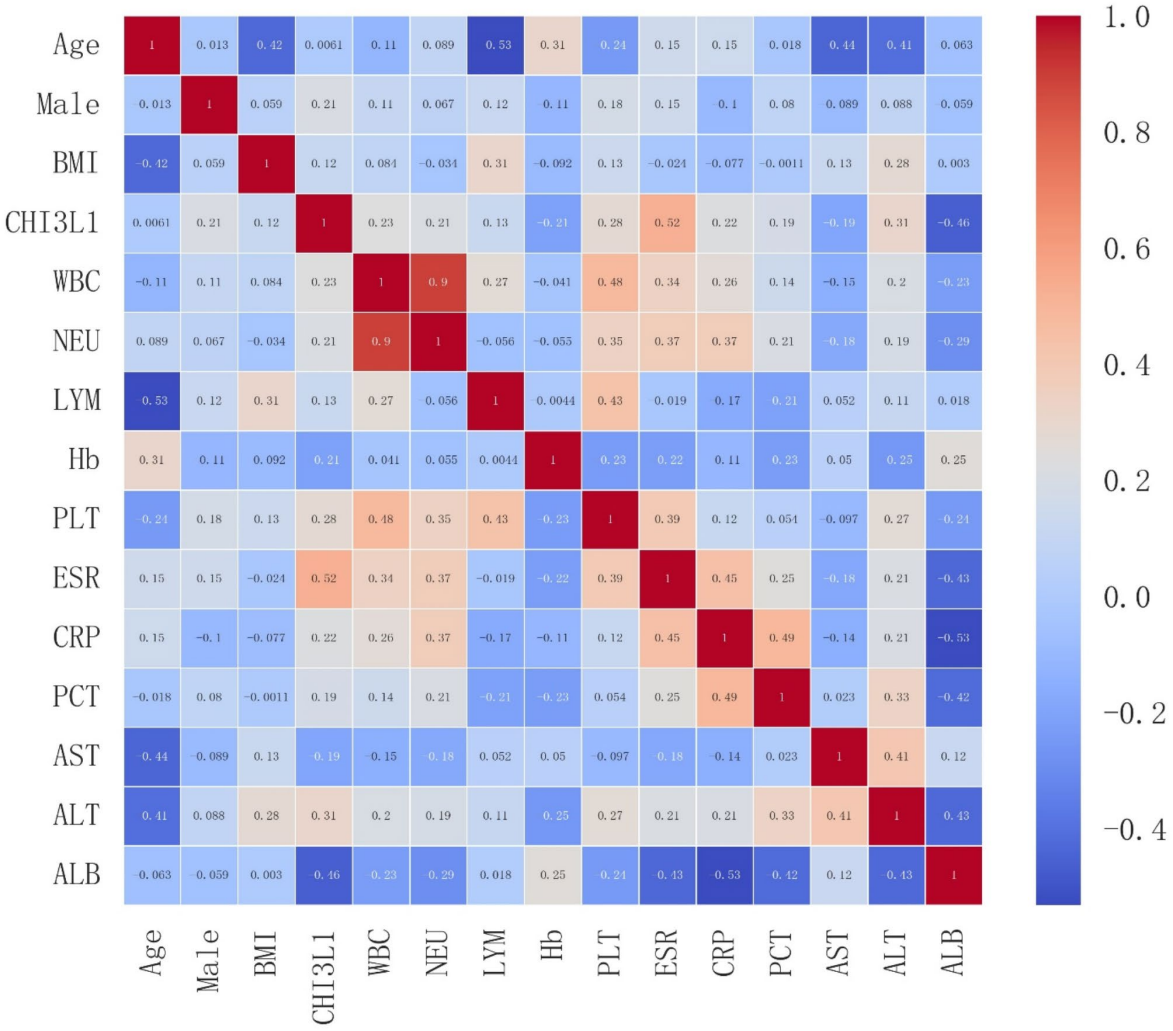
Correlation heat map between CHI3L1 and clinical parameters for KD.

The results presented in Fig. 3 illustrate a visual correlation heat map indicating that serum levels of CHI3L1 were positively correlated with male (*r* = 0.21, *p* = 0.011), WBC (*r* = 0.23, *p* = 0.004), NEU (*r* = 0.21, *p* = 0.009), PLT (*r* = 0.28, *p* < 0.001), ESR (*r* = 0.52, *p* < 0.001), CRP (*r* = 0.22, *p* < 0.001), and ALT (*r* = 0.31, *p* = 0.001). Conversely, serum levels of CHI3L1 were found to be negatively correlated with Hb levels (*r*=-0.21, *p* = 0.01), AST levels (*r*=-0.19, *p* = 0.018), and ALB levels (*r*=-0.46, *p* < 0.001).

### The association between CHI3L1 and KD

**Fig. 4:**
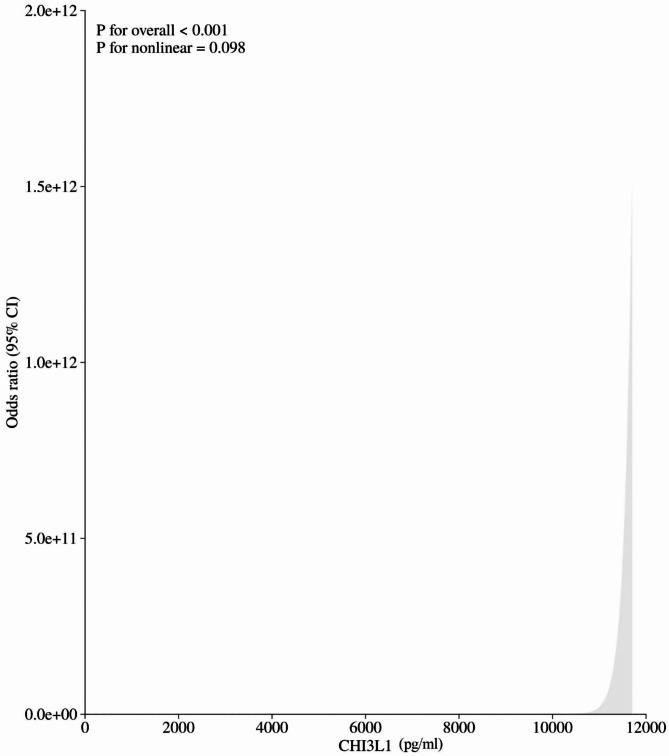
Odds ratio of KD according to serum of CHI3L1 levels in the overall population.

A univariate logistic regression analysis was initially conducted to identify potential factors associated with KD in order to avoid the effect of overfitting events. Variables with a significance level of *P* < 0.1 in the univariate analysis were subsequently included in a multivariate logistic regression model. Model 1, which was unadjusted, revealed a positive correlation between serum CHI3L1 levels and the presence of KD (OR = 216.0, 95% CI = 39.88-1170.03, p for trend < 0.001). Model 2 (Model 1 with adjusted for gender) (OR = 205.07, 95% CI = 37.76-1113.75, p for trend < 0.001) and model 3 (Model 2 with adjusted for WBC, NEU, Hb, PLT, ESR, CRP, ALT and ALB) (OR = 123.5, 95% CI = 10.13,1505.09, p for trend < 0.001) also showed a positive association of serum of CHI3L1 levels with the presence of KD (Table 3). As shown in Fig. 4, the RCS demonstrated a linear association between serum of CHI3L1 levels and the incidence of KD after adjustment for all confounders (p for non-linearity = 0.098).

**Table 3:** Association between CHI3L1 tertiles and the odds of KD.

Variables	Odds ratios, 95% CI and *P* value					
	Model 1	*p*-value	Model 2	*p*-value	Model 3	*p*-value
T1	Reference		Reference		Reference	
T2	10.57(3.59–31.06)	< 0.001	11.07(3.72–32.96)	< 0.001	15.81(2.13-117.34)	0.01
T3	216.0(39.88-1170.03)	< 0.001	205.07(37.76-1113.75)	< 0.001	123.5(10.13-1505.09)	< 0.001
p for trend		< 0.001		< 0.001		< 0.001

Model 1: adjust for none. Model 2: adjust for gender. Model 3: adjust for Model 2, WBC, NEU, Hb, PLT, ESR, CRP, ALT and ALB;

T1: CHI3L1 ≤ 2849 pg/ml, T2:2850 pg/ml < CHI3L1 < 6021 pg/ml; T3: CHI3L1 ≥ 6022 pg/ml.

### Potential diagnostic value of serum of CHI3L1 levels for KD

**Fig. 5:**
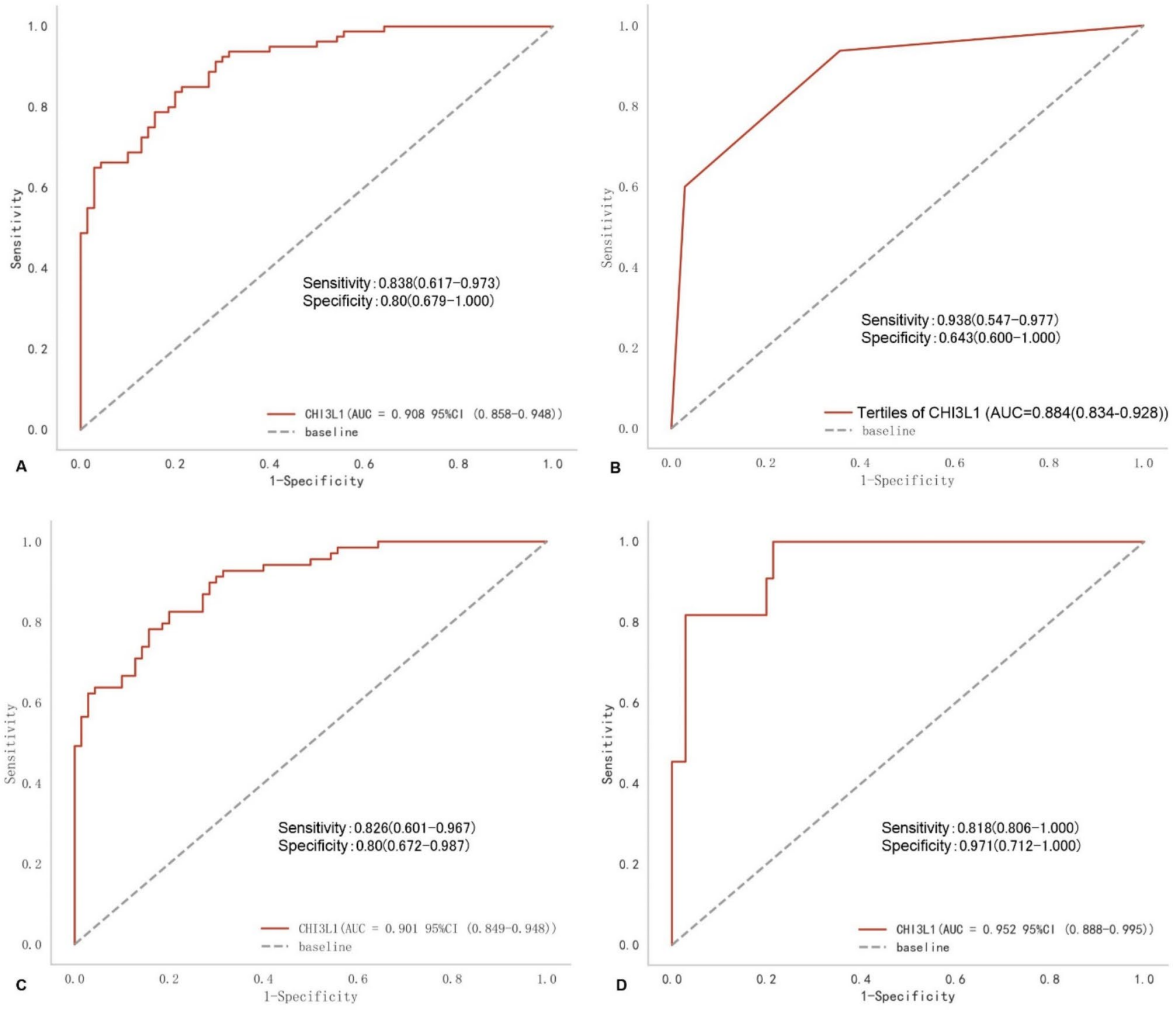
ROC curves of serum CHI3L to distinguish KD from febrile diseases. (**A**) predicting all types of KD; (**B**) The tertiles of CHI3L predicting all types of KD; (**C**) predicting cKD; (**D**) predicting iKD.

ROC curve analysis was utilized to assess the discriminatory ability of CHI3L1 in distinguishing KD from febrile illness (Fig. 5). The results indicated that continues CHI3L1 had an area under the curve (AUC) of 0.908 (with a cut-off value of 3720.0 pg/ml, sensitivity of 0.838, and specificity of 0.8) for differentiating all forms of KD(Fig. 5A). The tertiles of CHI3L1 exhibited significant predictive value for all types of KD, as evidenced by ROC curve areas of 0.884, a sensitivity of 0.938, and a specificity of 0.643 (Fig. 5B). Furthermore, continues CHI3L1 exhibited an AUC of 0.901, sensitivity of 0.826 and specificity of 0.8 for discriminating cKD at a cut-off of 3775.0 pg/ml (Fig. 5C), and an AUC of 0.952, sensitivity of 0.818 and specificity of 0.971 for discriminating iKD at a cut-off of 5773.8 pg/ml from febrile illness (Fig. 5D).

## Discussion

This study represents the first investigation into the diagnostic utility of serum CHI3L1 in KD. Our findings indicate elevated levels of CHI3L1 in both complete and incomplete Kawasaki disease, with a strong positive correlation between CHI3L1 levels and various inflammatory markers. These results suggest that serum CHI3L1 may serve as a promising novel biomarker for KD.

Early identification of KD in febrile illnesses is of paramount importance. Various KD scoring systems utilizing laboratory tests, clinical manifestations, or a combination of both have been developed to differentiate KD from other febrile diseases^3,17^. Furthermore, serological markers have also been explored as potential indicators for distinguishing KD from febrile illnesses^18,19^. Regrettably, a definitive and consistently reliable laboratory test for distinguishing KD from other febrile conditions has yet to be established. In this study, elevated serum of CHI3L1 levels were found to be significantly higher in patients with KD compared to both healthy controls and fever controls, consistent with previous findings from a study with a limited sample size^15^. Our investigation further demonstrated a positive correlation between serum of CHI3L1 levels and the presence of KD, which remained significant even after controlling confounders. RCS also showed a linear association between serum of CHI3L1 levels and the incidence of KD. Furthermore, the diagnostic accuracy of CHI3L1 as determined by the ROC curve indicated that both continuous and categorical CHI3L1 exhibited high sensitivity and specificity in distinguishing KD from other febrile illnesses. Similar findings were observed in neurodegenerative diseases^20^, liver fibrosis^21^, cardiovascular disease^11^, inflammatory diseases^22,23^. Previous studies have confirmed the diagnostic value of several biomarkers for KD, such as N-terminal pro-BNP^24^, serum ferritin^25^ and platelet-derived growth factor CC (PDGF-CC)^18^. N-terminal pro-BNP demonstrated a sensitivity of 0.89 and a specificity of 0.72 for the diagnosis of KD^24^. In contrast, PDGF-CC exhibited a sensitivity of 0.75 and a specificity of 0.76 for diagnosing KD^18^. Additionally, serum ferritin showed a sensitivity of 0.75 and a specificity of 0.83 in the diagnosis of KD^25^. Compared with these biomarkers, serum CHI3L1 demonstrates high sensitivity and specificity in both cKD (sensitivity: 0.826 and specificity: 0.8 and iKD (sensitivity: 0.818 and specificity: 0.971) in present study. This finding supports the notion that CHI3L1 may serve as a promising biomarker for KD. Pediatricians commonly encounter challenges in diagnosing iKD due to the absence of clear diagnostic criteria. Current findings demonstrate a significant elevation in serum CHI3L1 levels in both cKD and iKD patients compared to those with febrile illnesses. Additionally, in a mouse model of KD-like vasculitis, serum CHI3L1 levels were also found to be elevated^14^, which is in line with current results. CHI3L1 demonstrates satisfactory performance in distinguishing iKD and cKD from febrile diseases based on the ROC curve analysis. This data provides additional support for the diagnostic utility of CHI3L1 in KD.

Elevated levels of inflammatory cytokines such as interleukin-6 (IL-6), IL-20, interferon-γ (IFN-γ), and tumor necrosis factor-α (TNF-α)^26,27^, along with an increase in monocytes, macrophages, neutrophils^28,29^, and M1 macrophages^30^, have been implicated in the pathogenesis of KD. CHI3L1, a 40-kDa proinflammatory glycoprotein, plays a role in the pathophysiology of various conditions including asthma, inflammatory diseases^31,32^, cancers^33,34^, and cardiovascular diseases^32^. CHI3L1 has the ability to activate immune cells such as macrophages and neutrophils, leading to the production of proinflammatory cytokines such as IL-6, IL-8, and TGF-β^32,35,36^. Inhibition of CHI3L1 has been demonstrated to attenuate inflammation^37^. These findings suggest that increased levels of CHI3L1 may contribute to the release of inflammatory cytokines and activation of immune cells, potentially playing a role in the development of KD. Actually, our study showed that a positive association of serum CHI3L1 levels with inflammatory factors (i.e.,WBC, NEU, ESR, CRP), and negative association of serum CHI3L1 levels with Hb, AST and ALB in KD patients. Additionally, high CHI3L1 have the high incidence of KD, and high WBC, NEU, PLT, ESR, CRP and ALT, as well as lower Hb and ALB according to the tertiles of CHI3L1. Moreover, it has been established in prior studies that the upregulation of CHI3L1 can be induced by TNF-α, IL-6, and M1 macrophage conditioned media^38,39^. These results suggest that the interplay between CHI3L1 inflammatory factors and macrophages may play a role in the pathogenesis of KD. However, further investigation is required to validate this hypothesis.

It is important to note some limitations in our study, including its single-center design and the relatively small sample size, despite being larger than previous studies. Furthermore, the levels of CHI3L1 serum at various time points during the acute phase of KD were not taken into consideration. Finally, it is imperative to further investigate the potential mechanism of CHI3L1 in the pathogenesis of KD.

## Conclusion

Serum of CHI3L1 may be a novel and reliable biomarker for the diagnosis of KD.

## Data Availability

The datasets generated and/or analyzed during the current study are available from the corresponding author on reasonable request.
